# Effect of protein-rich diet and the tamarind trypsin inhibitor on behavioral disorders in obese zebrafish

**DOI:** 10.3389/fnut.2025.1706938

**Published:** 2026-01-20

**Authors:** Ana Karoliny Xavier de Gois Silva, Isaiane Medeiros, João Paulo Mamede, Aslan Costa Trajano, Thaiz Ivana da Silva Costa, Aline Lopes Marques de Sousa, Larissa Aida Lemos de Souza, Amanda Maria de Souza Nascimento, Juliana Kelly da Silva-Maia, Ana Heloneida de Araújo Morais

**Affiliations:** 1 Postgraduate Program in Nutrition,Department of Nutrition,Federal University of Rio Grande do Norte, Natal, Brazil; 2 Postgraduate Program in Biochemistry and Molecular Biology,Center for Biosciences,Federal University of Rio Grande do Norte, Natal, Brazil; 3 Postgraduate Program in Psychobiology,Center for Biosciences,Federal University of Rio Grande do Norte, Natal, Brazil; 4 Undergraduate Nutrition Course,Health Sciences Center,Federal University of Rio Grande do Norte, Natal, Brazil; 5 Department of Nutrition,Health Sciences Center,Federal University of Rio Grande do Norte, Natal, Brazil

**Keywords:** protease inhibitor, overfeeding, anxiety, social behavior, stress

## Abstract

**Introduction:**

Obesity is a major public health concern, often accompanied by behavioral and psychological alterations such as anxiety, impaired sociability, and dysregulated stress responses. Nutritional interventions using bioactive compounds from plant sources are emerging as promising strategies to address both metabolic and behavioral aspects of obesity. This study investigated the effects of tamarind trypsin inhibitor (TTI), a plant-derived bioactive protein, on behavioral and stress-related outcomes in diet-induced obese zebrafish (*Danio rerio*), a recognized translational model for human metabolic and neurobehavioral research.

**Methods:**

TTI was isolated and characterized by its molecular mass and enzymatic activity. Zebrafish were fed *Artemia* sp. at varying quantities, with or without TTI, to assess anxiety-like behavior, social behavior, and their response to acute stress. Obese overfed fish (OH) received 120 mg/fish/day, while eutrophic normofed fish (EN) received 75 mg/fish/day. Obese fish were treated for 10 days with TTI (25 mg/L), either while remaining overfed (OH+TTI) or becoming normofed (ON+TTI). On day 11, behaviors were recorded and analyzed using ANY-maze software.

**Results:**

Significant differences were found in anxiety-related average speed between OH and OH+TTI (*p* = 0.01), and between ON and OH+TTI (*p* = 0.01). Time spent at the top differed between EN and OH+TTI (*p* = 0.03), and immobility during stress was lower in OH+TTI compared to EN (*p* = 0.01). In the sociability test, average speed differed between EN and ON+TTI (*p* = 0.01).

**Discussion:**

Despite these findings, no consistent behavioral alterations indicating anxiety, sociability issues, or stress responses were associated with overfeeding or TTI. Thus, TTI did not induce behavioral side effects and may be a promising candidate for obesity treatment, differing from many current pharmacological options. Given the translational relevance of zebrafish to human nutrition and health, these findings highlight the potential of TTI as a candidate bioactive compound for future dietary interventions targeting both metabolic and behavioral outcomes in obesity.

## Introduction

Obesity is a major global health concern, associated with metabolic disorders, impaired quality of life, and increased risk of comorbidities such as diabetes, cardiovascular disease, and mental health disturbances (1–3).

Obesity is a multifactorial and complex disease, classified as pre-clinical, excess adiposity without current organ damage but with potential risk, or clinical, a chronic systemic inflammatory condition caused by excess fat leading to harm (1). Its severity involves interactions among lifestyle, genetic predisposition, hereditary, cultural, ethnic, and psychological factors (2, 3). Recent evidence shows psychological factors like compulsive eating, poor emotional regulation, and intense negative emotions, along with reduced physical activity, contribute to obesity development and are linked to psychological disorders and social issues, establishing a bidirectional relationship (4–6).

Another key factor in development of obesity is the quality and quantity of food consumed (2, 3). Dietary strategies, particularly those involving bioactive compounds from plant-based sources, have attracted attention as potential adjunctive therapies for obesity and related behavioral alterations. Ultra-processed foods, which are rich in energy density, sugars, saturated and trans fats, but low in fiber, protein, and essential nutrients, lead to high glycemic responses and low satiety due to additives and processing that alter the food matrix. These characteristics contribute to metabolic disturbances, gut microbiota imbalance, and low-grade inflammation, elevating the risk of obesity and systemic inflammation (7, 8).

Systemic inflammation in obesity can lead to neuroinflammation, affecting neurotransmitter production through neuroendocrine changes involving the hypothalamic-pituitary-adrenal (HPA) axis and inflammatory mediators (9, 10). The HPA axis also activates the stress system via increased cortisol and changes in the gut microbiota (9, 11). Therefore, obesity-related inflammation can cause behavioral changes through multiple pathways.

In the systematic review by Abiri et al. (12), the association between obesity phenotypes, metabolically healthy obesity (MHO), metabolically abnormal normal weight (MANW), and metabolically unhealthy obesity (MUHO), and psychiatric symptoms and quality of life was evaluated. The study found that individuals with MUHO had higher rates of depression, anxiety, and poorer quality of life compared to MHO individuals, highlighting the need for obesity treatment to be individualized based on each specific condition.

The management of MUHO obesity often requires therapeutic interventions (1). ABESO (3) recommends that treatment prioritize lifestyle changes, including nutritional guidance, physical activity, psychological support, and, when necessary, pharmacological or surgical approaches. In Brazil, ANVISA-approved medications for obesity include Sibutramine, Orlistat, Liraglutide, and Semaglutide. Notably, Semaglutide received approval from ANVISA in 2023 and from ABESO in partnership with the Brazilian Society of Endocrinology and Metabolism (SBEM) in 2024 for treating adults with obesity and associated comorbidities (12).

da Silva Valladares and Baiense (13) highlighted that certain obesity medications are associated with adverse behavioral effects, including outbursts, dependence, euphoria, schizophrenia, and delusions. Additionally, these drugs increase the risks of hypertension, cardiac issues, arrhythmias, psychological disorders, and hallucinations.

Alternative treatments for obesity, such as proteins, peptides, and enzymatic inhibitors with satiety-inducing effects, have been investigated. Tamarind (*Tamarindus indica*) seeds are a source of bioactive proteins, including a well-characterized trypsin inhibitor (TTI), which has demonstrated anti-inflammatory and metabolic modulatory effects in previous experimental models (14). However, little is known about its role in obesity-associated behavioral outcomes, such as anxiety, sociability, and stress reactivity.

Cristina Oliveira de Lima et al. (14), through an integrative review of 13 studies with animals and humans, reported consistent and beneficial effects of enzymatic inhibitors in promoting satiety. Among these, the trypsin inhibitor from tamarind seeds (TTI) stands out for its satiety-inducing and anti-inflammatory properties, showing potential for obesity treatment. Carvalho et al. (15) further emphasized TTI's multiple bioactive functions, including its role in preventing and treating obesity-related diseases.

The choice of TTI as the focus of this study was based on its unique biochemical profile compared with other plant-derived protease inhibitors. Previous studies in Wistar rats have shown that TTI reduces weight gain by decreasing food intake and increasing plasma cholecystokinin (CCK) levels (16). In addition to its satiety-inducing effects mediated by CCK modulation, TTI also exhibits potent anti-inflammatory activity in models of metabolic syndrome. Carvalho et al. (17) demonstrated that TTI significantly reduces plasma TNF-α levels in obese rats with diet-induced metabolic syndrome. These characteristics make TTI particularly relevant as a dual-action bioactive compound targeting both the metabolic and inflammatory pathways associated with obesity, supporting its selection over other inhibitors with more limited functional profiles. However, its effects on behavioral disorders related to obesity have not yet been evaluated, and despite toxicological safety data in rats and zebrafish (18, 19), its potential clinical application still requires caution.

In preclinical studies, zebrafish (*Danio rerio*) has emerged as a powerful translational model in nutritional and biomedical research, given its genetic and physiological similarities with humans, cost-effectiveness, and suitability for high-throughput analyses of metabolism and behavior. Studies in zebrafish have provided valuable insights into mechanisms of appetite regulation, energy metabolism, and stress responses that are relevant to human nutrition and disease (20, 21).

Zebrafish have become a valuable model for evaluating behaviors like anxiety, acute stress, and sociability due to their integrated nervous system and brain structures similar to mammals. Their well-characterized genetics and ability to develop obesity make them effective for studying metabolic dysfunctions (20–23).

Silva et al. (24) compared the effects of different diets on obesity and its complications in zebrafish. Fish fed a high-calorie, high-fat diet (egg yolk and oil) developed anxiety-like behavior, biochemical alterations, systemic inflammation, and organ damage, particularly in the intestines. Conversely, overfeeding with a healthier diet (*Artemia* sp.) also induced obesity and elevated inflammatory cytokines (TNF-α and IL-6), but triggered a milder organ inflammatory response and did not cause anxiety-like behavior (24).

Weight gain and obesity predisposition are risk factors for developing anxiety, or vice versa (25). However, few studies have examined how excess healthy diets induce obesity and affect behavioral disorders in zebrafish (24, 26). Therefore, the present study aimed to evaluate the effects of TTI supplementation on behavioral and stress-related outcomes in diet-induced obese zebrafish. By exploring the nutritional and neurobehavioral implications of this bioactive protein, our findings may contribute to the identification of novel dietary strategies with translational potential for the prevention and management of obesity and associated disorders in humans.

## Methods

### Obtaining and isolation of TTI

The tamarind fruit (*Tamarindus indica* L.) was acquired from a local market in Natal, Rio Grande do Norte, Brazil, identified by the Brazilian Institute of Environment and Renewable Natural Resources (IBAMA), Natal/RN (Brazil), and registered in the National System for the Management of Genetic Heritage and Associated Traditional Knowledge (SisGen) under number AF6CE9C. The extraction and characterization of TTI were conducted at the Laboratory of Chemistry and Function of Bioactive Proteins (LQFPB), Department of Biochemistry, UFRN (17).

For the preparation of the crude extract (CE), tamarind seeds were peeled, and the cotyledons were ground into a fine powder (sieved through a 40-mesh screen) using a refrigerated grinder at 6 °C. This flour was mixed with Tris-HCl buffer (50 mM, pH 7.5) in a 1:10 (w/v) ratio and stirred for 3 h at 22 °C. The mixture was then centrifuged (refrigerated centrifuge, Thermo Scientific, Sorval Legend XTR) at 10,000 rpm for 30 min at 4 °C, and the resulting supernatant was filtered, yielding the crude extract (CE). The CE was fractionated by ammonium sulfate precipitation into two saturation ranges: F1 (0–30%) and F2 (30–60%). Each precipitate was resuspended in 50 mM Tris-HCl buffer (pH 7.5), dialyzed, and stored at −20 °C in the same buffer.

The fraction with the highest inhibitory activity was purified by affinity chromatography using a Trypsin-Sepharose CNBr 4B column (10 cm × 1.5 cm) with 50 mM Tris-HCl buffer (pH 7.5). Aliquots of 5 mL were collected, and the retained proteins were eluted with 5 mM HCl, monitored at 280 nm by UV-visible spectrophotometry (DR 5000 Hach). The eluted proteins were dialyzed, lyophilized, and stored at −20 °C, obtaining TTI. The antitryptic activity was then determined using the N-benzoyl-DL-arginine-p-nitroanilide (BapNA) substrate (1.25 mM), following the method by Kakade et al. (26).

To quantify the proteins in TTI (14), bovine serum albumin was used at the following concentrations: 0.05–0.50 mg/ml. The determination was based on absorbance readings obtained using a microplate reader spectrophotometer (Biotek Epoch™ UV-Vis) at a wavelength of 595 nm (27). To assess the degree of isolation and estimate the molecular mass of proteins from EB, F1, F2, and TTI (28), the samples were subjected to SDS-PAGE using a 12.5% bisacrylamide gel. The samples were compared with the molecular weight marker Amersham™ ELCTM Marker—Full Range (GE Healthcare), with molecular weights of 225, 150, 102, 76, 52, 38, 31, 24, 17, and 12 kDa. The gel was silver-staining for protein visualization (29).

### Animals: maintenance and use

A total of 60 wild-type zebrafish (*D. rerio*) from the WT lineage were used, including 45 obese fish (average weight 0.752 g) and 15 eutrophic fish (average weight 0.568 g), all 4 months old and of both sexes. Classification was done using Body Condition Scoring charts based on lateral and dorsal views (30). All animals were sourced from the FishLab at UFRN, Brazil.

Obesity in zebrafish was induced by overfeeding, following Silva et al. (24) with modifications, using *Artemia* sp. biomass (from Artêmia Salina, RN). Over eleven weeks, fish received 60 to 120 mg wet weight per day, with weekly increases of 5.5 mg/fish, fed three times daily at 3-h intervals. In parallel, healthy eutrophic zebrafish were normofed with 15–75 mg/fish/day, also with weekly increases of 5.5 mg/fish, ensuring proper growth and development.

Zebrafish were kept under a 14h/10h light/dark cycle at 28 ± 1 °C, with constant monitoring of water quality parameters: oxygen (−7.8 mg/L), pH (7–8), nitrite (< 50 mg/L), and ammonia (up to 0.1 ppm) (31).

All experimental and animal care protocols were approved under protocol no. 035/2022 by the Ethics Committee on Animal Use of the Federal University of Rio Grande do Norte (CEUA-UFRN), following the guidelines of the National Council for the Control of Animal Experimentation (CONCEA, Brazil) and the ARRIVE guidelines (32), adhering to the 3Rs principles (Replacement, Reduction, Refinement) and OECD standards (33, 34).

### Experimental designer

All zebrafish were randomly allocated to groups using a random number generator to minimize selection bias. Each tank (*n* = 15 fish) was considered an independent experimental unit, and all behavioral analyses were performed by an observer blinded to group identity. Environmental variables such as light intensity (250–300 lux), water temperature (28 ± 1 °C), and testing time (between 9:00 and 13:00 h) were standardized across groups to ensure reproducibility. Although all behavioral evaluations were conducted using individual fish as experimental units, potential intra-tank dependencies were minimized by maintaining identical environmental and feeding conditions across tanks, as well as random assignment of fish.

That way, Zebrafish were divided into four groups of 15 fish, each housed in 12 L tanks, and fed 3 times daily at 3-h intervals for 10 days, per the specified experimental groups:

The study included four groups of 15 adult zebrafish each:

1. **Eutrophic normofed (EN)**, fed 75 mg of *Artemia* sp. biomass per fish/day without treatment;
2. **Obese overfed (OH)**, fed 120 mg/fish/day without treatment;
3. **Obese overfed treated with TTI (OH+TTI)**, fed 120 mg/fish/day, and treated with TTI at 25 mg/L;
4. **Obese normofed treated with TTI (ON+TTI)**, fed 75 mg/fish/day, and treated with TTI at 25 mg/L.

**Figure 1:**
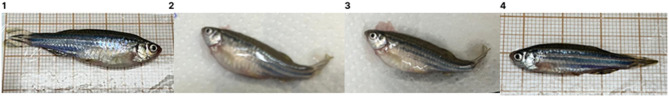
Images of zebrafish after 10 days of treatment with water or TTI. 1: Eutrophic normofed without treatment. 2: obese overfed without treatment. 3: obese overfed treated with TTI. 4: obese normofed treated with TTI. Image source: Author's own.

All animals were removed from their home tanks and placed in an exposure tank containing either water (control) or TTI (experimental) for 1 h before feeding. Afterward, they returned to their original tanks and waited 20 min before receiving food. Experimental groups were transferred to separate tanks (same size and volume as the originals) and exposed to TTI at 25 mg/L, diluted in filtered water, and added dropwise. This procedure was repeated daily for 10 days (Figure 1).

The TTI concentration (25 mg/L) was selected based on prior studies in adult zebrafish that reported no acute or subchronic toxicity and demonstrated consistent biochemical activity at similar or lower concentrations (18). This dose ensured sufficient exposure to elicit biological effects while maintaining animal welfare standards. Control (EN and OH) and treatment (ON+TTI and OH+TTI) groups were balanced for sex and body condition score, considering the inherent characteristics of each group to avoid confounding effects related to sex-dependent or size-associated behavioral differences.

On the 11th day, fish were subjected to behavioral tests: novel tank test (anxiety-like behavior), shoaling preference (social behavior), and acute stress test (alarm substance exposure). Behaviors were recorded with a Logitech HD webcam and analyzed using ANY-maze software (Stoelting Co., version 6.33).

### Social behavior test: shoaling preference test

The social preference test, commonly used to assess zebrafish social behavior, leverages their natural shoaling tendency. It is highly sensitive to pharmacological treatments that affect social preference similarly to effects seen in humans and rodents (35, 36).

Three rectangular tanks (20 cm × 16 cm × 12 cm), each containing 2.8 L of water, were arranged side by side. One fish from each experimental group was placed in the central tank, separated from the lateral tanks, which contained random shoals of the same-age zebrafish not involved in the experiment. Opaque barriers prevented visual contact between the central fish and the shoals.

The behavior of each experimental fish was recorded for 5 min, first with white panels in place, then for another 5 min after removing the panels, testing one fish at a time. The central tank was virtually divided into three vertical zones (proximal to the shoal, middle, and distal), with the area closest to the social stimulus as the focus for analysis using Any-maze software. Parameters evaluated included time spent in the social area, total distance traveled, average speed, and latency to first entry into the shoal area.

### Anxiety test: novel tank test

Locomotor activity and anxiety-related behaviors were assessed using the novel tank diving test, following Fontana et al. (35). Fifteen fish from each experimental group were individually placed in a rectangular tank (20 cm × 16 cm × 12 cm) filled with 2.8 L of water, with sides covered by white paper except the front for video recording. Each fish's behavior was recorded for 5 min immediately after being introduced to the new tank.

The aquarium was virtually divided into three horizontal zones (top, middle, and bottom, each 4 cm high) to assess locomotor activity and anxiety-like behaviors. Anxiety was induced by placing fish in a new tank, with anxiety-like behavior identified by their preference for the bottom zone (33). The evaluated parameters included total distance traveled, average swimming speed, time immobile, distance traveled at the bottom, and time spent at the top (37, 38).

### Acute stress test: exposure to conspecific alarm substance (CAS)

Some fish species store an alarm substance in epidermal cells, which, when released into the water after a predator-induced injury, triggers defensive responses in other fish of the same species (39).

**Figure 2:**
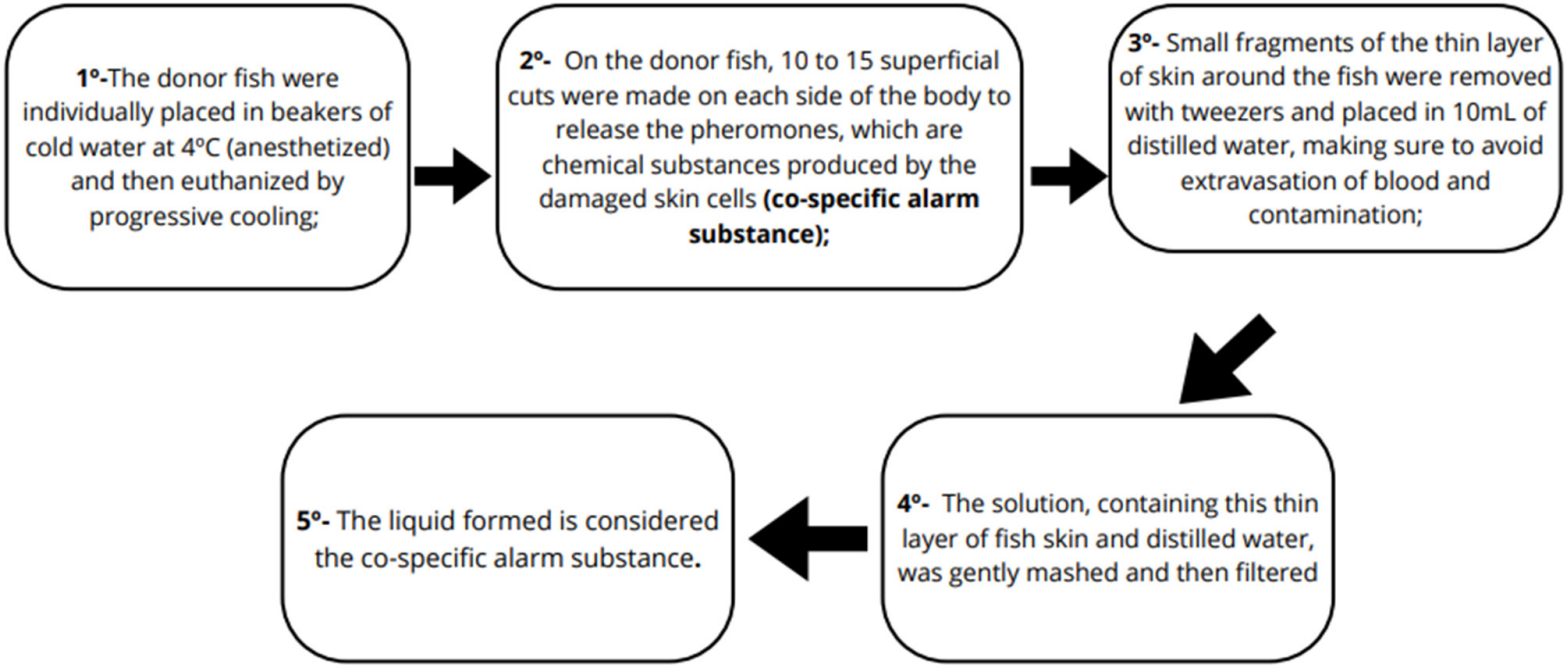
Schematic representation showing the preparation protocol for the alarm substance used in the acute stress experiment.

This study induced acute stress in zebrafish by extracting conspecific alarm substance from donor fish outside the experimental groups. The experiment aimed to assess stress response intensity differences between adequately fed and overfed fish. The preparation of the alarm substance followed specific procedures involving the donor fish (Figure 2).

The conspecific alarm substance was prepared and extracted entirely in chilled distilled water to preserve the delicate skin layers, using 6 donor fish from a random shoal not involved in the experimental groups.

For acute stress assessment, 15 fish from each experimental group were individually placed in rectangular tanks (20 cm × 16 cm × 12 cm) with 2.8 L of water, sides covered with white paper except the front. After acclimation, baseline behavior was recorded for 5 min. Then, 1 mL of conspecific alarm substance was added using a 1000 μl pipette, and behavior was filmed for another 5 min.

Video recordings were analyzed using ANY Maze software, with the tank divided into three horizontal zones (top, middle, bottom). The evaluated parameters were average speed, immobility time, distance traveled at the bottom, and time spent at the top. Comparisons were made between the initial time (before alarm substance exposure), and final time (after exposure).

The exposure time and concentration of the alarm substance were selected based on previous studies showing consistent aversive behaviors in zebrafish (40, 41). Results were analyzed by calculating the variation between initial (pre-exposure) and final (post-exposure) times, averaging these values per group and parameter to obtain a single value for statistical analysis.

### Statistical analysis

Because the behavioral data did not meet the assumptions of normality, as confirmed by visual inspection and formal statistical tests, non-parametric Kruskal–Wallis analyses followed by Dunn's *post hoc* comparisons were applied. This approach aligns with standard practices in zebrafish behavioral research, where interindividual variability frequently results in non-Gaussian data distributions (35). Control groups consisted of eutrophic normofed and obese overfed zebrafish without treatment, and comparisons were performed across all experimental and control groups. Analyses were conducted using GraphPad Prism version 10 (USA).

Although no formal power analysis was performed, the number of animals per group was similar to or greater than that used in comparable zebrafish behavioral studies, suggesting adequate sensitivity to detect large effects. Effect sizes and multiple-comparison considerations were incorporated into the interpretation of results to ensure robust and transparent conclusions. The global Kruskal–Wallis effect size was η^2^ = 0.093. Multiple comparisons were corrected using the Benjamini–Hochberg false discovery rate (FDR) procedure.

## Results

### Isolation and characterization of tamarind trypsin inhibitor (TTI)

**Figure 3:**
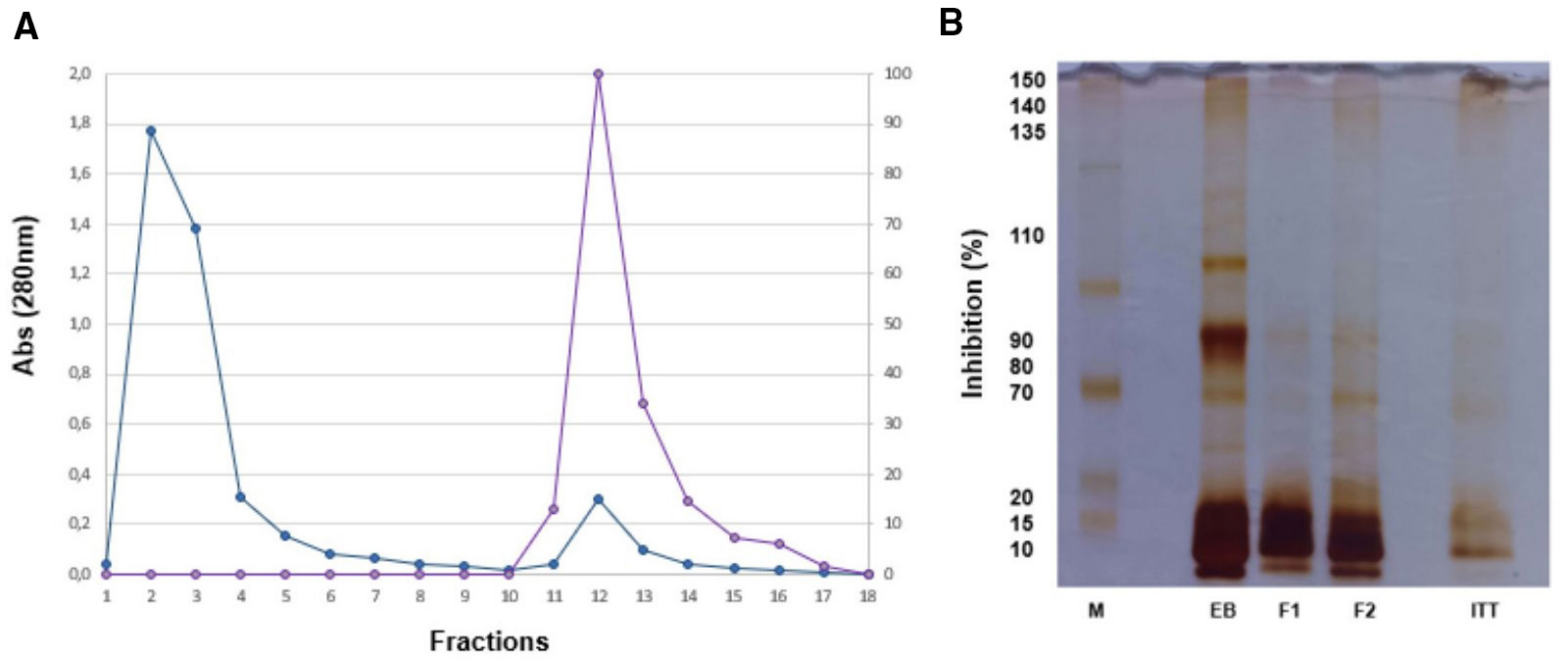
Chromatographic Profile of F2 and Estimation of TTI Molecular Mass. **(A)** The chromatographic profile of F2, subjected to trypsin-Sepharose 4B affinity chromatography, involved pre-equilibration with 50 mM Tris-HCl buffer (pH 7.5). Unretained proteins (blue line) were eluted with the same buffer, while retained proteins (purple line) were eluted with 5 mM HCl. Aliquots of F2 (5.0 ml) were monitored at 280 nm. The inhibitory activity of the second protein peak was evaluated using a trypsin inhibition assay with 100 μl (0.7 mg) of TTI and BAPNA at 1.25 mM as substrate. **(B)** Additionally, SDS-PAGE at 12.5% under denaturing conditions, stained with silver nitrate, was performed to visualize proteins from the molecular weight marker (M), crude extract (CE, 10 μg/ml), fraction F1 (0–30% ammonium sulfate, 10 μg/ml), fraction F2 (30–60% ammonium sulfate, 10 μg/ml), and isolated TTI (5 μg/ml).

After isolating TTI through Trypsin-Sepharose CNBr 4B affinity chromatography, it was found that 0.7 mg of TTI inhibited trypsin activity by 96.61% (408.22 IU/mg). SDS-PAGE (12.5%) under denaturing conditions, stained with silver nitrate, confirmed TTI isolation with a predominant molecular mass of approximately 20 kDa (Figure 3).

### Social behavior test: schooling preference test

**Figure 4:**
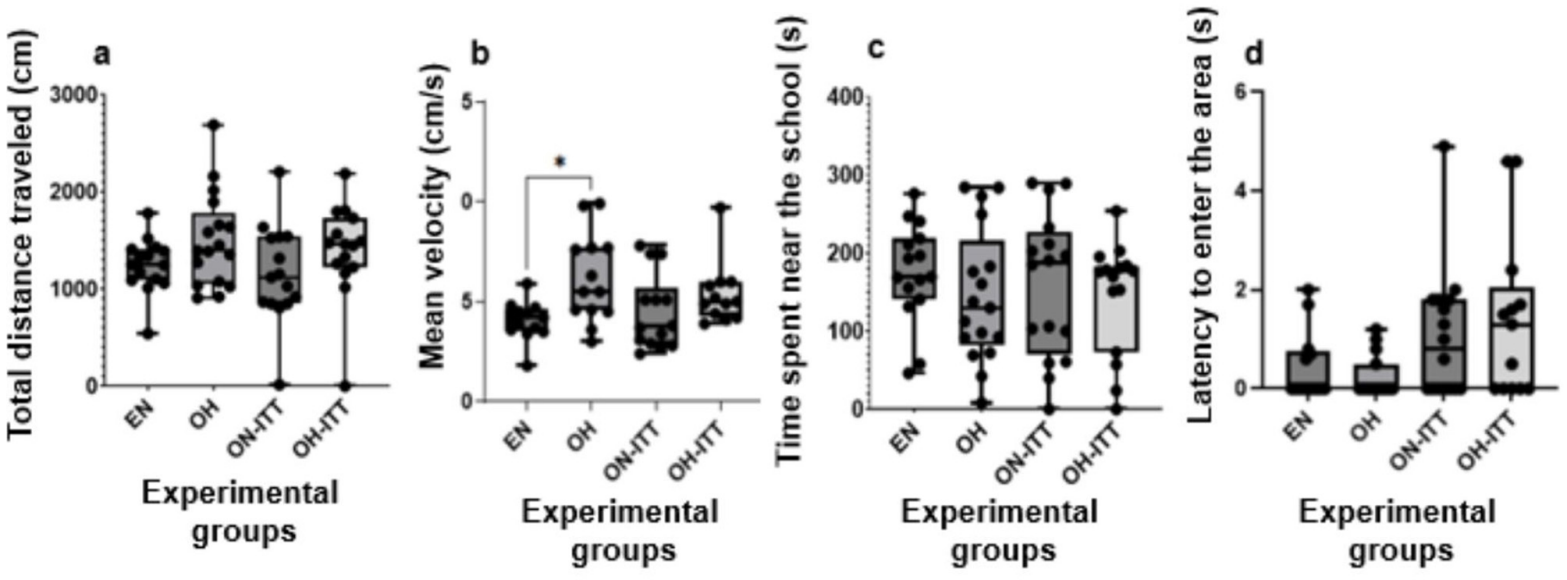
Sociability behavior of zebra fish in the schooling preference test eutrophic normofed zebra fish without treatment (EN, *n* = 15) and obese overfed zebra fish without treatment (OH, *n* = 15) were considered control groups. The animals in the treatment groups were: obese normofed treated with TTI for 10 days (ON+TTI, *n* = 15) and obese overfed treated with TTI for 10 days (OH+TTI, *n* = 15). At the end of the treatment period, the animals from all four groups were placed in a tank with a group of conspecifics beside them for social interaction assessment over 6 min. **(a)** Total distance traveled in the tank, **(b)** average swimming speed, **(c)** time spent near the school, and **(d)** Latency to enter the school area. *Indicates significant differences between groups (Kruskal–Wallis, *p* < 0.05).

In the sociability test, the Kruskal–Wallis analysis showed no significant differences in distance traveled between zebrafish groups (χ^2^ = 5.81, *p* = 0.12; Figure 4a). However, average speed differed significantly (χ^2^ = 10.53, *p* = 0.01; Figure 4b), with the obese overfed group (OH) displaying higher velocity than the eutrophic normofed group (EN) (*p* < 0.05), though no differences were found with other groups. Time spent near the shoal (χ^2^ = 1.73, *p* = 0.63; Figure 4c) and latency to enter the shoal area (χ^2^ = 7.39, *p* = 0.06; Figure 4d) showed no significant differences across groups.

### Anxiety test: novel tank test

**Figure 5:**
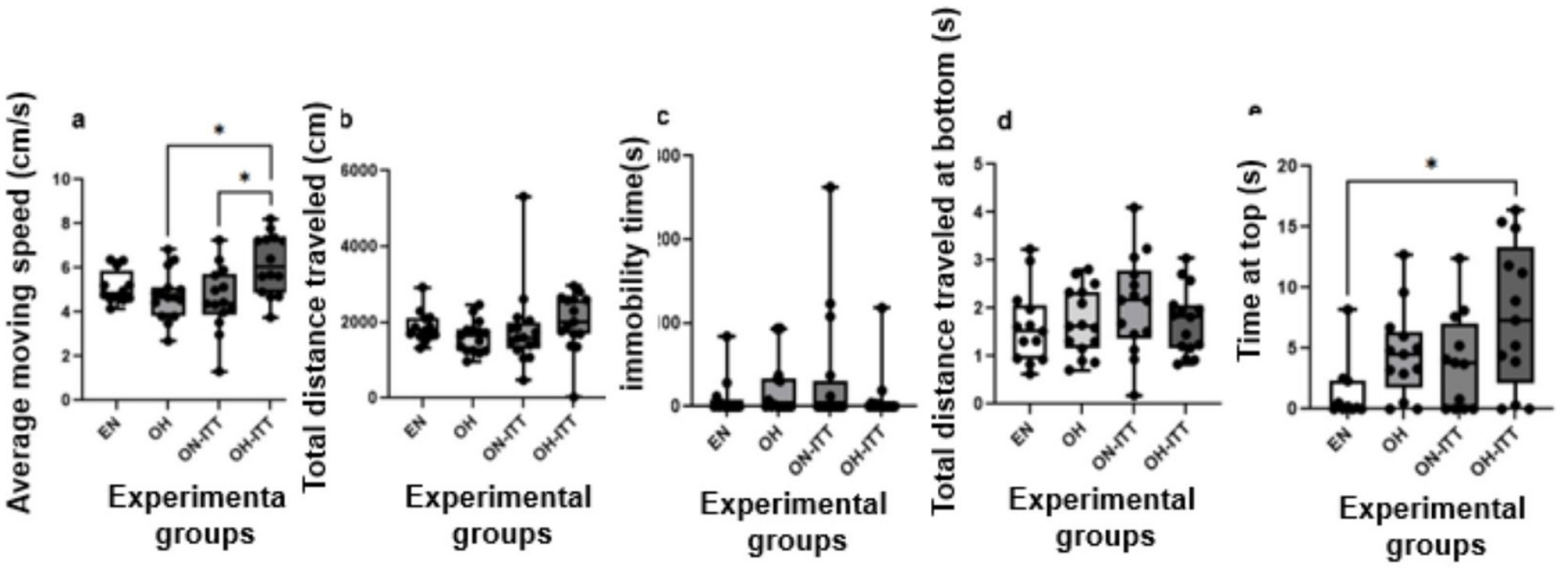
Anxiety behavior of zebra fish in the novel tank test acclimated for 5 minutes. Eutrophic normofed zebra fish without treatment (EN, *n* = 15) and obese overfed zebra fish without treatment (OH, *n* = 15) were considered control groups. The treatment groups were: obese normofed zebra fish treated with TTI for 10 days (ON+TTI, *n* = 15) and obese overfed zebra fish treated with TTI for 10 days (OH+TTI, *n* = 15). At the end of the treatment period, animals from all 4 groups were placed in a novel tank, and their behavior was filmed for 6 min. **(a)** Average swimming speed, **(b)** total distance traveled, **(c)** immobility time (freezing), **(d)** distance traveled at the bottom of the tank, and **(e)** time spent at the top of the tank. Indicates significant differences between groups (Kruskal–Wallis, *p* < 0.05).

The anxiety-like behavior in zebrafish was evaluated using the novel tank test and analyzed with the Kruskal–Wallis test^**^. A significant difference was observed in the average swimming speed among the groups (χ^2^ = 10.63, d*f* = 3, *p* = 0.01). *Post hoc* Dunn's test revealed that obese overfed zebrafish treated with TTI (OH+TTI) exhibited higher average speed compared to obese normofed zebrafish treated with TTI (ON+TTI) and obese overfed zebrafish without treatment (OH) (*p* < 0.05). No significant differences were found between the other groups (Figure 5a).

The analysis of zebrafish locomotion showed no significant differences between groups for total distance traveled (χ^2^ = 6.38, *p* = 0.09; Figure 5b), immobility time (χ^2^ = 1.22, *p* = 0.75; Figure 5c), time spent at the bottom of the tank (χ^2^ = 3.43, *p* = 0.488), or distance traveled at the bottom (χ^2^ = 2.26, *p* = 0.51), indicating no anxiety-like behavioral changes among the groups (Figure 5d).

Finally, the time spent by zebrafish at the top of the tank showed significant differences between groups (χ^2^ = 9.16, *p* = 0.03). *Post hoc* analysis revealed that obese overfed zebrafish treated with TTI (OH+TTI) spent more time at the top compared to eutrophic normofed zebrafish without treatment (EN) (*p* < 0.05), with no differences observed among the other groups (Figure 5e).

### Acute stress test: exposure to co-specific alarm substance (ESAC)

**Figure 6:**
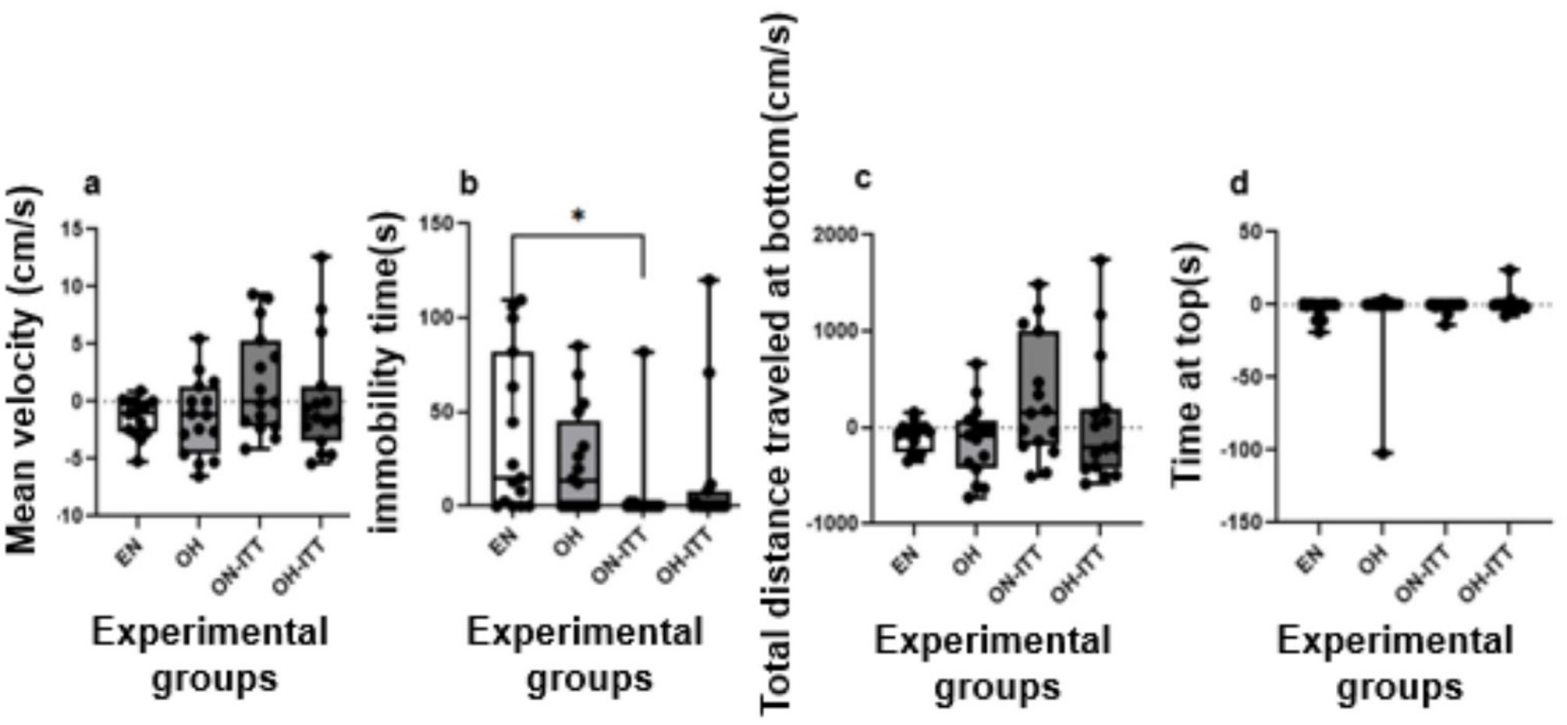
Acute stress behavior of zebrafish in the alarm substance exposure test, 5 Min before and 5 Min after. Eutrophic normofed zebrafish without treatment (EN, *n* = 15) and obese overfed zebrafish without treatment (OH, *n* = 15) were considered control groups. The treated groups included: obese normofed zebrafish treated with TTI for 10 days (ON+TTI, *n* = 15) and obese overfed zebrafish treated with TTI for 10 days (OH+TTI, *n* = 15). At the end of the treatment period, the animals from all four groups were placed in a tank and filmed for 5 min before and 5 min after the introduction of the alarm substance. The behavioral results presented are the difference in each animal's behavior before and after the alarm substance was introduced into the tank. Therefore, positive values indicate an increase in behavior after the presence of the alarm substance, and negative values indicate a decrease in behavior. **(a)** Mean swimming speed, **(b)** immobility time, **(c)** distance traveled in the bottom area, and **(d)** time spent in the upper area of the tank. *Indicates a significant difference between groups (Kruskal–Wallis, *p* < 0.05).

The zebrafish response to the alarm substance, assessed via the Kruskal–Wallis test, showed no significant differences in mean swimming speed among the groups (χ^2^ = 3.19, d*f* = 3, *p* = 0.36; Figure 6a), indicating similar speeds under all tested conditions.

Regarding fear/anxiety behaviors, immobility time showed significant differences between groups (χ^2^ = 11.64, *p* = 0.01), with the eutrophic normofed group (EN) remaining immobile longer than the normofed obese zebrafish treated with TTI (ON+TTI) (*p* < 0.05), though no differences were found with other groups (Figure 6b). Distance traveled at the bottom (χ^2^ = 3.53, *p* = 0.32; Figure 6c) and time spent at the top (χ^2^ = 2.14, *p* = 0.54; Figure 6d) showed no significant differences between the groups.

All statistical comparisons include corresponding χ^2^ and *p* values, which are now presented in the Supplementary Table S1.

## Discussion

This study assessed the effects of overfeeding, with or without TTI treatment, a protein studied as a potential obesity therapy, on behavioral disorders in adult obese zebrafish. Notably, the diet used is nutritionally balanced for this model when given in proper amounts.

*Artemia* sp. is a crustacean-based animal food offered at different developmental stages, like newly hatched or 48-h nauplii and adult biomass. It is a key protein source for aquatic animals and widely used as live feed for crustacean and fish larvae. Providing live *Artemia* in tanks benefits fish by stimulating their natural predatory behavior through hunting (37). In this study, frozen *Artemia* biomass was thawed and fed to zebrafish in excess, protein-rich diet (120 mg wet weight/fish/day) for 11 weeks, leading to weight gain and obesity. Obese fish treated with TTI and/or normofed (75 mg/fish/day) were compared to obese and eutrophic fish without TTI using behavioral tests, including the novel tank test (anxiety), social preference test (sociability), and alarm reaction test (acute stress).

Although this study did not observe behavioral disorders related to anxiety, stress, or sociability in obese zebrafish, whether normo or overfed with *Artemia* sp., and with or without TTI, other studies point to a strong association between obesity and anxiety in zebrafish (9, 24, 42–45).

Our findings indicate that TTI supplementation did not negatively influence in anxiety-like behavior, sociability, and stress responses in obese zebrafish. These results are consistent with the growing evidence that dietary bioactive proteins may be positive not only for metabolic but also for behavioral and neuroendocrine outcomes. Importantly, zebrafish have been increasingly validated as a translational model for human obesity and stress-related disorders, given the conservation of neuroendocrine pathways such as the HPA axis and neurotransmitter systems involved in anxiety and social interaction.

In this study, anxious-type behavior was assessed using the novel tank test. A significant difference was observed in the average movement speed parameter (Figure 4a) between the obese overfed zebrafish group and the obese overfed fish treated with TTI, as well as between the obese normofed fish and the obese overfed fish treated with TTI. Additionally, a significant difference was found in the time spent at the top parameter (Figure 5e) between the eutrophic normofed untreated fish and the obese overfed fish treated with TTI, with a significant increase for the latter group.

In the novel tank test, two of five parameters showed that overfeeding combined with TTI caused behavioral changes. However, these fish only differed from eutrophic normofed fish in time spent at the top, which indicates reduced anxiety and behavior within the normal range. This suggests the need to assess additional behavioral aspects to better understand the effects of diet and TTI.

In the sociability test, a significant difference was observed in average velocity between eutrophic normofed zebrafish without treatment and obese overfed fish without treatment (Figure 4b), suggesting that overfeeding may increase locomotion speed. However, among the four evaluated parameters, this was the only one affected, and TTI showed no influence on sociability behavior.

Other studies suggest obesity is linked to altered social behavior in animals (34, 43, 45, 46). Zebrafish, known for their strong preference for same-species groups (47), exhibit social behavior through stereotyped swimming patterns directed toward their group. This behavior is controlled by forebrain neurons that may be homologous to mammalian brain regions involved in social interactions (48). Although no differences in social behavior were observed, this is expected since obesity is linked to neuroinflammation and behavioral changes like anxiety and depression, highlighting obesity's impact on emotional health (49). However, the diet used (*Artemia* sp.) is nutritionally adequate and ideal for zebrafish, even when given in excess, which may explain the absence of behavioral disorders despite obesity in the animals.

In this study, under acute stress induced by alarm substance, a significant difference in immobility time (Figure 6b) was observed between eutrophic normofed fish and obese normofed fish treated with TTI. TTI reduced immobility time in this group, indicating a behavioral change in only one parameter. Therefore, only TTI in obese normofed fish appeared to reduce the acute stress response, as obese overfed fish showed no differences, with or without TTI, in any of the four parameters evaluated.

Moreover, obesity induced by overfeeding with *Artemia* sp. caused significant weight gain, biochemical and inflammatory changes, and hepatic steatosis, but did not trigger anxious-type behavior. In contrast, a high-fat diet (egg yolk with soybean oil) led to intestinal inflammation and anxiety-like behavior, although only one behavioral test was used (24). Silva et al. (24) also highlighted that differences in diet nutrient quality influence the secondary effects of obesity, with consequences varying in intensity depending on diet composition, individual biochemistry, and factors such as gender.

Rakhra et al. (50) showed that even nutritionally balanced foods like *Artemia* sp., when consumed excessively, can cause weight gain and fat-related complications. Other studies confirm that overeating healthy foods can also lead to obesity issues (50). Similarly, Anwer et al. (45) found that zebrafish fed an obesogenic diet (live *Artemia* sp. and dry feed) displayed greater behavioral changes, including exploratory behavior in anxiety tests and reduced social interaction, with more intense responses in aversive learning, personality traits, and anxiety assays.

Commercial feeds, commonly used in experimental studies, have been evaluated for their nutritional composition and physiological, biochemical, histopathological, and behavioral effects (24). These feeds often contain excessive carbohydrates compared to zebrafish's needs and, even when properly dosed, can trigger significant inflammatory responses, as shown by Silva et al. (24). Thus, the behavioral effects observed in Ghaddar et al. (51) may also be influenced by the composition of the dry feed used alongside *Artemia* sp.

Excess digestible carbohydrates in the zebrafish diet are stored as triglycerides and, when dietary fat is insufficient, their oxidation to meet energy demands can significantly affect anxiety levels (23). Picolo et al. (43) showed that a high-fat diet increased aggressiveness, induced anxiety-like behavior, and impaired memory formation. However, in the novel tank diving test, no changes in locomotion or vertical exploration were observed (43), suggesting that diet type and composition may influence behavioral alterations differently. Similarly, the systematic review by Abiri et al. (12) found that humans with obesity and metabolic disorders are more likely to develop depression, anxiety, and have a lower quality of life compared to those with metabolically healthy obesity.

In another zebrafish study, a high-fat diet increased expression of genes linked to inflammation and apoptosis, damaging brain and liver tissues, while also altering anxiety and aggression behaviors (22). Additionally, obesity-related anxiety may involve neurotransmitter system changes; for example, increased hippocampal TNF-α levels due to obesity-induced inflammation contributed to anxiety-like behaviors in both fish and mice (52).

Obesity has been linked to the accumulation of senescent glial cells near the lateral ventricle, impairing neurogenesis and promoting anxiety in obese mice (53). High-fat diets also disrupted GABAergic neurotransmission in the dorsomedial hypothalamus, contributing to anxiety-like behaviors in rats (50). Additionally, the gut-brain axis may play a key role in obesity-related cognitive and mood disorders, as inflammation, often resulting from microbiota dysbiosis, can affect the HPA axis, alter neuroactive metabolites, and promote neuroinflammation (50).

In a previously mentioned study, overfeeding with *Artemia* sp. to induce obesity increased inflammatory mediators like TNF-α and IL-6, although it did not cause intestinal inflammation compared to other diets (24). This inflammatory response could influence the behavioral disorders examined in the present study. However, the level of inflammation in obesity appears to affect its link with behavioral disorders differently. The gut-brain axis, especially in relation to inflammation, seems to play an important role in the development of these disorders, as highlighted by Agustí et al. (54).

Studies show that zebrafish fed high-fat diets or overfed with egg yolk and commercial feed display anxiety-like behaviors, highlighting that excessive intake of certain macronutrients like fats and carbohydrates can strongly affect emotional behaviors by promoting inflammation and other effects linked to poor diet quality (43, 55). These findings emphasize the complex relationship between obesity, anxiety, and related disorders, reinforcing that, in addition to physiological factors such as gender and age (24, 56), consumption of pro-inflammatory foods and nutrients is a key factor in the development of these behavioral disorders.

This study did not detect anxiety-, stress-, or sociability-related behavioral disturbances in obese zebrafish, whether they were normally fed or overfed with *Artemia* sp., and regardless of TTI treatment. One possible explanation for the absence of consistent behavioral alterations involves adaptive or compensatory mechanisms triggered by chronic overfeeding. Prolonged nutritional excess may attenuate stress reactivity by inducing hypothalamic pituitary interrenal (HPI) axis hyporesponsiveness, as described by Yehuda et al. (56). Such adaptation could mask anxiety-like or stress-related phenotypes even under conditions of metabolic imbalance. Alternatively, sustained overfeeding may itself act as a chronic stressor, resulting in behavioral habituation and diminished responsiveness to acute challenges (54).

Some commonly used obesity medications have been linked to behavioral side effects, especially anxiety. Sibutramine, for example, can cause insomnia, high blood pressure, tachycardia, headaches, anxiety, and irritability (57). Orlistat also carries risks, including reduced absorption of fat-soluble vitamins and potential worsening of conditions like cardiac arrhythmias and psychotic episodes. It may also cause anxiety, increased blood pressure, heightened myocardial contractility, and even chemical dependency (58).

Previous studies in rodent models have demonstrated that plant-derived trypsin inhibitor (TTI) can reduce food intake, attenuate weight gain, and modulate inflammatory responses. TTI is known for inducing satiety (19), reducing inflammation (17), and improving blood sugar control (59). Previous research confirmed TTI's safety in zebrafish embryos and adults (18). However, its impact on obesity-related behavioral disorders had not been studied before this work.

Our results extend these observations by showing their effects in an aquatic vertebrate model. These cross-species findings reinforce the hypothesis that TTI may exert conserved effects relevant to human health, particularly in the context of obesity and its psychological comorbidities. This study evaluated the effects of an excess diet, nutritionally ideal for zebrafish but given in excess, on behaviors like anxiety, stress, and sociability, alongside TTI, a protein from tamarind seeds with bioactive properties (15).

The hyperactivity observed in one group of obese zebrafish treated with TTI in the novel tank test, although significantly higher than in untreated obese overfed fish, was not considered relevant, as it appeared to be an isolated finding. Similarly, the hyperactivity seen in the obese overfed group during the social interaction test was not supported by differences in distance traveled or social behavior parameters. Thus, neither overfeeding nor TTI induced consistent behavioral disorders.

Few studies have explored fear responses to acute or chronic stressors in individuals with obesity. This study used zebrafish as a model due to their ability to exhibit fear/anxiety responses in ecologically relevant situations, such as exposure to alarm substances. These substances trigger typical fear behaviors like erratic movements, darting, and freezing (60, 61), mediated by olfactory sensory neurons and involving brain regions such as the telencephalon, lateral habenula, and locus coeruleus (61).

Although extrapolation from zebrafish to humans requires caution, the present study supports the view that zebrafish can provide valuable insights into the nutritional modulation of behavior and stress. The observed effects of TTI suggest that this bioactive protein warrants further investigation in mammalian models and, ultimately, in clinical trials. If confirmed in humans, TTI supplementation could contribute to the development of innovative dietary strategies for obesity management, targeting both metabolic and behavioral dimensions of the disease.

Importantly, although TTI did not induce adverse behavioral effects in this short-term model, the study design does not allow conclusions about long-term neurobehavioral safety or potential metabolic interactions. Thus, these findings should be interpreted as preliminary and limited to the specific conditions evaluated. In addition, because no tissue-level exposure markers (e.g., CCK, cortisol, and TNF-α) were measured, internal dosing was inferred solely from the administered concentration. Future studies should incorporate biochemical or molecular biomarkers to confirm systemic uptake and elucidate the mechanistic actions of TTI. Research integrating behavioral, inflammatory, and metabolic endpoints in mammalian models is also warranted to further validate these observations.

A limitation of our study is that it did not directly assess metabolic endpoints such as glucose or lipid profiles, which would provide a more integrated picture of TTI's effects. Future research should address these aspects and explore the molecular pathways underlying the behavioral outcomes observed. Nevertheless, the current findings provide a strong rationale for advancing TTI research into more complex models of human nutrition and obesity. In addition to further investigating the possibility that the absence of behavioral changes may be attributed to chronic stress and hyporesponsiveness resulting from prolonged overfeeding.

Additionally, the apparent differences in average swimming speed across groups may reflect locomotor variability rather than genuine emotional modulation. This distinction reinforces the importance of combining behavioral assays with physiological markers (e.g., hormones, cytokines) to disentangle motor influences from affective components.

In this study, the fear response was evaluated in obese zebrafish, showing no differences between overfed and normofed groups (Figure 6). A significant reduction in immobility (freezing) was observed only in normofed obese fish treated with TTI (Figure 6b); however, this effect cannot be attributed to TTI, as previous toxicity studies reported no influence of TTI on fear/anxiety responses (18). While further studies in mammalian models and clinical settings are warranted, our findings provide preliminary evidence supporting the translational relevance of zebrafish for nutritional research and suggest that TTI could represent a promising candidate for future dietary interventions aimed at obesity management and related behavioral disturbances in humans.

## Conclusion

In conclusion, under the experimental conditions of this study, overfeeding zebrafish with *Artemia* sp., a protein-rich and nutritionally balanced diet when properly dosed, induced significant weight gain and obesity without causing consistent changes in anxiety-, sociability-, or stress-related behaviors, regardless of TTI treatment. These findings indicate that TTI did not produce behavioral side effects in this short-term model, supporting its compatibility with zebrafish physiology and suggesting a preliminary safety profile in obese organisms. However, because *Artemia* sp. is not a pro-inflammatory diet and this model does not address long-term neurobehavioral or metabolic outcomes, conclusions regarding the broader safety or translational relevance of TTI should remain cautious. Future studies using pro-inflammatory overfeeding paradigms and integrating behavioral, neuroinflammatory, and metabolic endpoints in both zebrafish and mammalian models are needed to clarify how TTI interacts with metabolic imbalance and to better define its potential as an adjunct strategy in obesity management.

## Data Availability

The original contributions presented in the study are included in the article/Supplementary material, further inquiries can be directed to the corresponding author/s.
