# Supplementary material for: Effect of protein-rich diet and the tamarind trypsin inhibitor on behavioral disorders in obese zebrafish
Source: Front Nutr. 2026 Jan 20;12:1706938. doi: 10.3389/fnut.2025.1706938 (PMC12864065; doi:10.3389/fnut.2025.1706938)
Supplement: Supplementary file 1 [file Table_1.docx]

# Supplementary

# Table S1. Statistical results for behavioral tests in obese zebrafish exposed to a protein-rich diet and Tamarind Trypsin Inhibitor (TTI).

| Behavioral Test | Variable Analyzed | Statistical Test | χ² (df) | p-value | Interpretation |
| --- | --- | --- | --- | --- | --- |
| Sociability test | Mean swimming speed | Kruskal–Wallis | χ² = 5.81 (3) | p = 0.12 | OH > EN (higher locomotion) |
| Sociability test | Distance in social area | Kruskal–Wallis | χ² = 10.53 (3) | p = 0.01 | No significant difference |
| Sociability test | Time in social area | Kruskal–Wallis | χ² = 1.73 (3) | p = 0.63 | No significant difference |
| Sociability test | Latency to enter social zone | Kruskal–Wallis | χ² = 7.39 (3) | p = 0.06 | No significant difference |
| Novel tank test | Mean swimming speed | Kruskal–Wallis | χ² = 10.63 (3) | p = 0.01 | OH+TTI > ON+TTI and OH |
| Novel tank test | Time in upper zone | Kruskal–Wallis | χ² = 6.38 (3) | p = 0.09 | OH+TTI > EN (trend only) |
| Novel tank test | Immobility time | Kruskal–Wallis | χ² = 1.22 (3) | p = 0.75 | No significant difference |
| Novel tank test | Spent at the bottom of the tank | Kruskal–Wallis | χ² = 3.43 (3) | p = 0.488 | No significant difference |
| Novel tank test | Time spent at the top | Kruskal–Wallis | χ² = 9.16 (3) | p = 0.03 | OH+TTI > EN (more time at the top) |
| Novel tank test | Distance in bottom zone | Kruskal–Wallis | χ² = 2.26 (3) | p = 0.51 | No significant difference |
| Alarm substance test | Mean swimming speed | Kruskal–Wallis | χ² = 3.19 (3) | p = 0.364 | No difference |
| Alarm substance test | Immobility time | Kruskal–Wallis | χ² = 11.64 (3) | p = 0.009 | EN > ON+TTI (increased freezing) |
| Alarm substance test | Distance to bottom | Kruskal–Wallis | χ² = 3.53 (3) | p = 0.317 | No difference |
| Alarm substance test | Time at surface | Kruskal–Wallis | χ² = 2.14 (3) | p = 0.543 | No difference |

Legend: EN = normofed control; ON = normofed + TTI; OH = overfed control; OH+TTI = overfed + TTI. All pairwise comparisons performed using Dunn’s post hoc test; adjusted p-values via Benjamini–Hochberg correction.
